# Risk model of hepatocellular carcinoma based on cuproptosis-related genes

**DOI:** 10.3389/fgene.2022.1000652

**Published:** 2022-09-15

**Authors:** Zhiqiang Liu, Yong Qi, Haibo Wang, Qikun Zhang, Zhengsheng Wu, Wenyong Wu

**Affiliations:** ^1^ Department of General Surgery, The First Affiliated Hospital of Anhui Medical University, Hefei, China; ^2^ Department of Pathology, The First Affiliated Hospital of Anhui Medical University, Hefei, China

**Keywords:** liver cancer, cuproptosis, molecular subtype, risk score, prognosis, tumor immunity

## Abstract

**Background:** Owing to the heterogeneity displayed by hepatocellular carcinoma (HCC) and the complexity of tumor microenvironment (TME), it is noted that the long-term effectiveness of the cancer therapy poses a severe clinical challenge. Hence, it is essential to categorize and alter the treatment intervention decisions for these tumors.

**Materials and methods:** “ConsensusClusterPlus” tool was used for developing a secure molecular classification system that was based on the cuproptosis-linked gene expression. Furthermore, all clinical properties, pathway characteristics, genomic changes, and immune characteristics of different cell types involved in the immune pathways were also assessed. Univariate Cox regression and the least absolute shrinkage and selection operator (Lasso) analyses were used for designing the prognostic risk model associated with cuproptosis.

**Results:** Three cuproptosis-linked subtypes (clust1, clust2, and clust3) were detected. Out of these, Clust3 showed the worst prognosis, followed by clust2, while Clust1 showed the best prognosis. Three subtypes had significantly different enrichment in pathways related to Tricarboxylic Acid (TCA) cycle, cell cycle, and cell senescence (*p* < 0.01). The clust3 subtype with poor prognosis had a low “ImmuneScore” and low immune cell infiltration, and the three subtypes had significant differences in the antigen processing and presentation pathway of the macrophages. Clust1 had a low TIDE score and was sensitive to immunotherapy. Then, according to the prognosis-related genes of cuproptosis, a prognosis risk model related to cuproptosis was constructed, containing seven genes (KIF2C, PTTG1, CENPM, CDC20, CYP2C9, SFN, and CFHR3). “High” group had a higher TIDE score compared to the TIDE score value shown by the “Low” group, which benefited less from immunotherapy, whereas the “High” group patients were more sensitive to the conventional drugs. Finally, the prognosis risk model related to cuproptosis was combined with clinical pathological characteristics to further improve the prognostic model and survival prediction.

**Conclusion:** Three new molecular subgroups based on cuproptosis-linked genes were revealed, and a cuproptosis-related prognostic risk model comprising seven genes was established in this study, which could assist in predicting the prognosis and identifying the patients benefit from immunotherapy.

## Introduction

Liver cancer includes primary liver cancer and secondary forms of liver cancer. Hepatocellular carcinoma (HCC) is a very prevalent type of primary liver cancer, followed by intrahepatic cholangiocarcinoma and other rare cancers (sarcoma, hemangioendothelioma, etc.) (Li et al., 2022). Liver cancer shows a poor prognosis. The frequency of liver cancer has significantly increased in the past few years, while its 5-year survival OS rate is <20% (Pham et al., 2022). Hepatitis B and C viruses, non-alcoholic fatty liver disease (NAFLD), alcohol consumption, and other factors (i.e., aflatoxin and microcystin) are among the primary causes of liver cancer. Out of these, HBV and HCV are seen to be the major risk factors for liver cancer (Lin et al., 2020). Although numerous high- or low-expression genes linked to the onset of liver cancer and carcinogenesis have been identified, the probable molecular mechanism of liver cancer is not entirely understood. Precision medicine can introduce a fresh perspective for individualized cancer diagnosis and focused therapy by considering the heterogeneity of every patient. Therefore, clinicians should propose more specific diagnosis and treatment methods for the subtype of the disease for optimizing the efficacy of treatment, thereby decreasing the resulting side effects (Liang et al., 2018).

From bacteria and fungi to plants and animals, copper is the basic element of life. In the human body, it combines with enzymes to help blood clots, hormone maturation, and cell energy processing and is also involved in many biological behaviors. However, too much copper will kill cells and cause pathological damage to multiple organs. Studies have shown that copper ion is both a key cofactor of many enzymes, and excessive copper ion will lead to cell death. The exact mechanism of cuproptosis involves the induction of cell death after combining the tricarboxylic acid cycle (TCA)-linked enzymes, leading to a protein toxic stress response, which differs from the cell death mechanisms discussed in the past (Tsvetkov et al., 2022). Many studies have shown that copper metabolism is involved in many pathophysiologies of chronic hepatitis. A long-term exposure to a higher concentration of copper ions or the long-term usage of unqualified copper water pipes and tableware could lead to chronic copper poisoning, thereby causing chronic liver disease (Guo et al., 2021; Nakaichi et al., 2021). In addition, cuproptosis has aroused widespread concern in a variety of liver diseases. Excessive copper exposure can lead to oxidative stress, due to excessive reactive oxygen species (ROS) production and reduced antioxidant function, and then promote hepatocyte apoptosis through mitochondrial apoptosis. Earlier reports also stated that the TNF-R1 signaling pathway played a vital role in the Cu-induced apoptosis pathway (Liu et al., 2020). Copper metabolism is closely related to human-related genetic disease hepatolenticular degeneration (Xu et al., 2021), and such patients have been associated with copper storage disorders for a long time. Hence, it becomes important to determine novel molecular markers and identify the cuproptosis-linked downstream signaling pathways, for understanding the regulatory role played by cuproptosis in the pathophysiology of liver cancer.

In this report, the cuproptosis-linked genes were used to identify stable molecular subtypes through consistent clustering. Thereafter, the clinical characteristics, pathway characteristics, and immune characteristics were compared between the different subtypes. Finally, genes related to the cuproptosis phenotype were detected using the expression difference analysis and least absolute shrinkage and selection operator (Lasso). Furthermore, the risk model and clinical prognostic model were constructed, which can assist in the personalized treatment of liver cancer patients.

## Materials and methods

### Data collection and processing

The Cancer Genome Atlas (TCGA) GDC API was used for downloading TCGA-LIHC dataset containing RNA-seq data, copy number variation (CNV) and mutation data used in this study. Primary tumor samples were remained. Samples with no survival information were removed. After the screening, 50 normal and 360 primary tumor samples were included in this study. The Gene Expression Omnibus (GEO) database provided the gene expression data for the GSE14520 dataset. Following identification, 242 liver carcinoma samples were used in the study. Here, the TCGA-LIHC was used as a training set, while the GSE14520 dataset was used as an independent verification set. The cuproptosis-linked genes in this study came from the study of Tsvetkov et al. (2022), a total of 13 cuproptosis-related genes, i.e. ATP7A, LIAS, LIPT1, DLD, DBT, DLST, FDX1, PDHA1, DLAT, GCSH, PDHB, SLC31A1, and ATP7B. The bioinformatics analysis of this study was supported by the Sangerbox tool (http://vip.sangerbox.com/) (Shen et al., 2022). The work flow of this study was shown in Supplementary Figure S1.

### Data preprocessing

The RNA-seq data downloaded from the TCGA database were preprocessed as mentioned below: 1) All samples without any clinical follow-up data were discarded; 2) All samples without information regarding their survival duration were eliminated; 3) All samples without their OS were eliminated; 4) Ensembl was converted to the Gene symbol; and 5) Median values of the expressions with multiple gene symbols were considered. On the other hand, the GEO data were pre-processed as follows: For the GEO data set, the annotation information of the corresponding chip platform was downloaded. According to the annotation information, the probe was mapped to the gene, and the probe that matched multiple genes was discarded. If a gene matched multiple probes, the median value was regarded as its gene expression value.

### Molecular subtypes of the cuproptosis-linked genes

ConsensusClusterPlus was used for consistent clustering to build a consistency matrix, and the samples were clustered and typed (Wilkerson and Hayes, 2010). The molecular subtypes of all the samples were derived using the expression data of the cuproptosis-linked genes. “Pam” algorithm and “Euclidean” were used as the distance measurement, and 500 bootstraps were conducted, wherein every bootstrap process included 80% of all patients in a training set. The cluster number was defined as between 2 and 10, and the best classification was selected by determining the consistency matrix and consistency cumulative distribution function for determining the molecular subtype of the sample.

### Constructing a risk model

1) Through the molecular subtypes identified previously, the cuproptosis-linked genes with differences between the subtypes were identified. Here, the differences between the clust1 vs. non-clust1 subtypes, clust2 vs. non-clust2, and clust3 vs. non-clust3 subtypes, were identified through the Limma package (Ritchie et al., 2015). The differentially expressed genes (DEGs) were also identified based on their FDR<0.05 and | log2FC |>1 values.
2) Univariate Cox analysis was conducted through the Cox function in the survival package, and DEGs with significant prognosis (| logFC |>1 & FDR<0.05) were selected.
3) Lasso regression (Tibshirani, 1997) was used to decrease the number of genes. Stepwise regression was then utilized, using the Akaike Information Criterion (AIC), which considered the model’s statistical fit and the no. of parameters that could be used for fitting. The most complex model was used to start the stepAIC technique in the MASS package (Zhang, 2016), and one variable was eliminated at a time to lower AIC. The model performed better with a smaller value, indicating that it had achieved an acceptable degree of fit with fewer parameters.

The RS of each patient was estimated using the formula as follows: RiskScore (RS) = Σβ_i_ × EXP_i_. EXP_i_ refers to the gene expression level of gene characteristics related to the prognosis of cuproptosis-related phenotypes, while β_i_ refers to a Cox regression coefficient for the respective gene. To categorize patients into high-risk and low-risk RS groups, survminer R package (http://www.sthda.com/english/rpkgs/survminer/) was used to calculate the optimal cut-off. KM curve was used for drawing the survival curve for prognostic analysis, while the log-rank test was employed for determining the significant difference between the groups.

### Gene set enrichment analysis technique

For investigating the pathways associated with various biological processes in numerous molecular subtypes, the “GSEA” technique was utilized for pathway analysis (Subramanian et al., 2005). Here, GSEA was analyzed using the c2. cp.kegg.v7.0. symbols.gmt as a background set through GSEA software, and identified with NP < 0.05. In addition, the TCA cycle-associated genes and pathways were downloaded from the MSigDB database in GSEA (http://www.GSEa-msigdb.org/GSEa/msigdb/search.jsp), and the ssGSEA was used for calculating the score of the TCA related pathways. Then, the pathways and genes related to cell growth and death were downloaded from KEGG’s official website (https://www.kegg.jp/kegg/pathway.html), and the score of the cell growth and death-related pathways was calculated by ssGSEA. In addition, the NK Cytotoxicity Score, Toll-Like Receptor Score, and the Antigen Processing and Presentation Score for every sample were determined using the ssGSEA process, with the help of the relevant genes involved in these pathways, derived from the GSEA-based MSigDB database.

### Calculation of invasion abundance of tumor microenvironment cells

The relative abundance of the 22 immune cells involved in lung cancer was measured using the CIBERSORT algorithm (https://cibersort.stanford.edu/) (Newman et al., 2015). Simultaneously, the percentage of immune cells was determined using the Estimation of Stromal and Immune Cells in Malignant Tumors Using Expression Data (ESTIMATE) software, and the Wilcoxon rank sum test was employed for comparing the degree of immune cell infiltration between the high-risk and the low-risk groups (Runa et al., 2017).

### Prediction of responsiveness to immunotherapy

The effect of the Immune Checkpoint Inhibitor Score (IMS) on predicting the Immune Checkpoint inhibitors’ (ICI) clinical reactivity was confirmed using the Tumor Immune Dysfunction and Exclusion (TIDE) algorithm. Immune Checkpoint Blockade (ICB) reactivity is predicted using the gene expression profile by the TIDE algorithm (Jiang et al., 2018). The TIDE algorithm assessed two distinct mechanisms of the tumor immune escape scores, such as tumor-infiltrating Cytotoxic T Lymphocytes (CTLs) dysfunction score (dysfunction) and the immunosuppressive factor rejection score (exclusion), as well as 3 cell types that restricted T cell infiltration into the tumors, such as M2 subtype of the cancer-associated fibroblasts (CAF), myeloid-derived suppressor cells (MDSCs), and the tumor-associated macrophages (TAMs). The potential clinical consequences of immunotherapy in the new molecular subgroups were assessed in this study using the TIDE software (http://tide.dfci.harvard.edu). The likelihood of immunological escape increases with increasing TIDE prediction score, indicating that patients are less likely to benefit from immunotherapy.

## Results

### Gene mutations and transcriptional changes of cuproptosis-related genes

**FIGURE 1:**
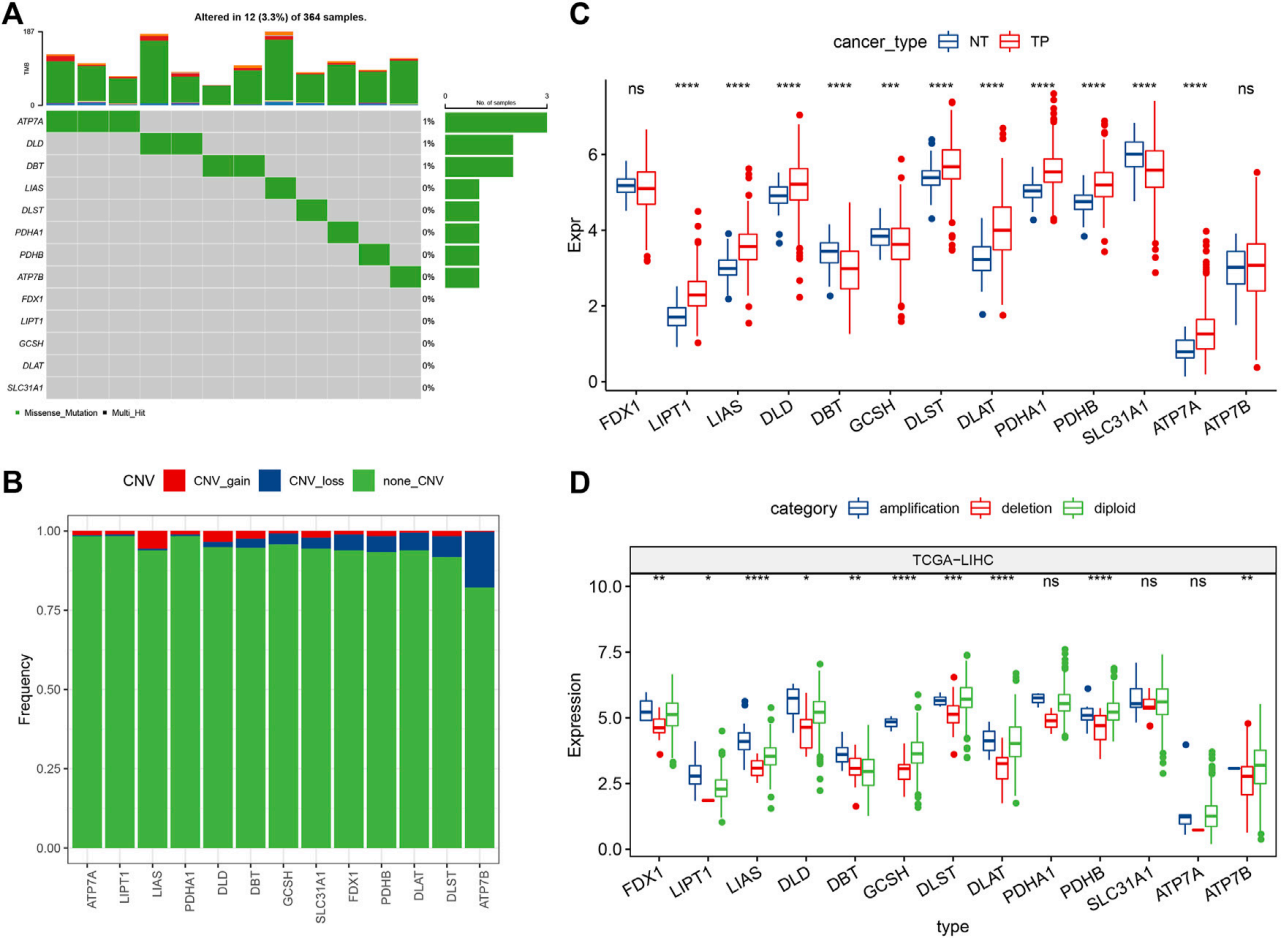
Mutation map and expression characteristics of cuproptosis-related genes in liver cancer. **(A)** Mutation map of cuproptosis-linked genes in the primary tumor samples; **(B)** CNVs of cuproptosis-linked genes in the primary tumor samples. Vertical axis indicates the percentage of CNV types of cuproptosis-related genes; **(C)** The differences of gene expression levels between different CNV types in primary tumor samples; **(D)** Differential analysis of transcriptional expression levels of cuproptosis-linked genes in primary tumor and adjacent normal tissue samples. Log2 (expression) was selected in C and D. ns, not significant. *p < 0.05, **p < 0.01, ***p < 0.001, ****p < 0.0001.

In this study, 13 cuproptosis-related genes were obtained. For determining the genetic changes caused by “cuproptosis” in liver cancer, the gene mutation rate of the somatic mutations in 13 cuproptosis genes was evaluated. Among 364 TCGA-LIHC primary tumor samples, 12 (3.3%) had mutations in cuproptosis-linked genes (Figure 1A). Among them, only ATP7A, DLD, and DBT had gene mutations. Then, we analyzed the somatic copy number changes of these cuproptosis-related genes in primary liver cancer and found that cuproptosis-related genes had a low CNV amplification/deletion frequency (Figure 1B). To determine if the genes related to cuproptosis are differentially expressed in primary tumors and normal tissues, the mRNA changes of cuproptosis-linked genes between the primary tumor samples and the adjoining normal tissue samples were compared, showing that a majority of the cuproptosis-linked genes were differentially expressed (Figure 1C). Further, to explore the difference in CNV value in mRNA expression in primary tumor tissues, patients with primary liver cancer were categorized into 3 groups according to CNV value, including increased CNV, CNV loss, and no significant change in CNV. Then, the mRNA expressions of the cuproptosis-linked genes between all groups were compared (Figure 1D). The results indicated that most of these cuproptosis-linked genes showed higher expression in patients with increased CNV and patients with lost CNV, and there was no significant change compared with CNV.

### Molecular typing depending on the cuproptosis-linked genes

**FIGURE 2:**
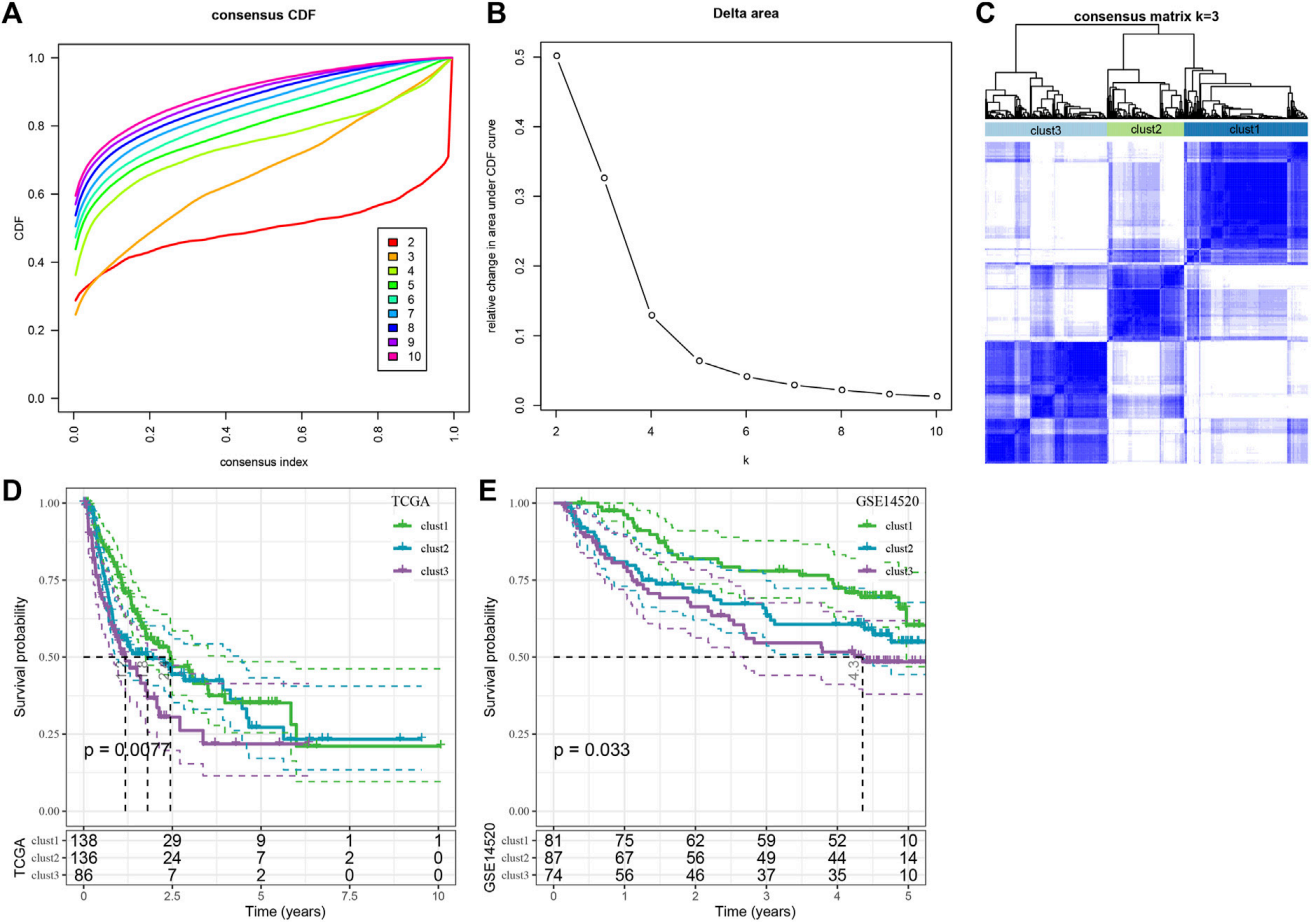
Consensus clustering analysis based on the prognosis of cuproptosis-linked genes in liver cancer. **(A)** CDF curve of TCGA-LIHC dataset samples; **(B)** CDF-delta area curve for the TCGA-LIHC dataset, Delta area curve for consensus clustering, which indicates the relative difference in the area under the CDF curve for every category number, k, in comparison to the k—1. The *X*-axis axis denotes the category number, k, whereas the *Y*-axis indicates the relative change in the area under the CDF curve; **(C)** Sample clustering-related heat map when the consumption *k* = 3; **(D)** KM curves denoting the correlation between the prognosis of 3 subtypes, identified using the TCGA-LIHC dataset; **(E)** KM curve of prognosis of three subtypes in GSE14520 cohort.

For understanding the expression pattern of the cuproptosis-linked genes, the liver cancer samples in the TCGA-LIHC dataset containing clinical information were used to classify patients through the consistent clustering of the expression profiles of these 13 cuproptosis-related genes. Then, an optimal no. of clusters was determined based on the cumulative distribution function (CDF), and the CDF Delta area curve showed that if the selected cluster was 3, it showed a very stable clustering outcome (Figures 2A,B), Finally, the k-value of 3 was selected to determining 3 molecular subtypes (Figure 2C). Analysis of the prognostic features of the 3 molecular subtypes showed that they displayed significant prognostic differences (Figure 2D). It was noted that clust3 showed the worst prognosis, followed by clust2, and clust1 had the best prognosis. This same technique was used for verifying the GSE14520 dataset and the results showed that significant differences existed in the prognosis of the 3 molecular subtypes (Figure 2E), which was in agreement with the TCGA-LIHC dataset.

### Clinical characteristics and mutation characteristics between molecular subtypes

The clinicopathological characteristics of the numerous molecular subtypes in the TCGA-LIHC dataset were assessed. Then, the distribution of various clinical characteristics in 3 molecular subtypes was compared, and all distribution differences in the clinical characteristics of different subtypes were determined. The results revealed significant differences between the clust1 and clust2/clust3 in T-stage, Stage, and Grade (Supplementary Figure S2A). T1/T2 accounted for a relatively high proportion in clust1/clust2, and T3/T4 accounted for an increase in clust3. In the stage distribution, T1/T2 accounted for a relatively high proportion in clust1/clust2, and T3/T4 accounted for an increase in clust3. In the grade distribution, the proportion of clust1 in G1/G2 was relatively high, and the proportion of clust2/clust3 in G3/G4 was increased.

### Mutation characteristics between the molecular subtypes

**FIGURE 3:**
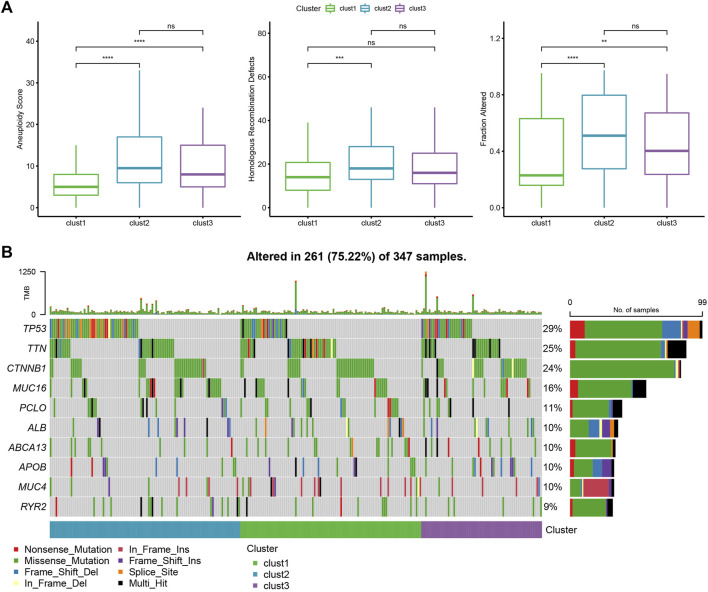
Genome changes of molecular subtypes in the TCGA-LIHC dataset. **(A)** The differences among the molecular subtypes of the TCGA-LIHC dataset were compared in terms of Aneuploidy Score, Homologous Recombination Defects, Fraction Altered. **(B)** The top 10 mutated genes in three subtypes. *p < 0.05; **p < 0.01; ***p < 0.001; ****p < 0.0001.

The different genomic changes noted in the 3 molecular subtypes existing in the TCGA-LIHC dataset were analyzed. Here, the molecular characteristics of TCGA-LIHC were downloaded from an earlier Pan-cancer Study (Thorsson et al., 2018). It can be seen that the clust1 subtype showed a low Aneuploidy Score and Homologous Recombination Defects (Figure 3A). In addition, a previous study divided HCC into 5 molecular subtypes according to 160 immune signatures, of which the immune molecular subtypes C1, C2, and C4 had the worst prognosis and C3 showed the best prognosis. In a comparison of the relationship between the 5 immune molecular subtypes and the proposed 3 molecular subtypes, it was noted that the C1/C2/C4 subtype of immune molecular subtype occupied more in clust3 and clust2 subtypes with poor prognosis, while the C3 subtype of immune molecular subtype occupied more in clust1 subtype with good prognosis (Supplementary Figures S2B,C). In addition, the differences in the gene mutations in various molecular subtypes were compared. TP53 was the mostly mutated gene in all three subtypes, with a total mutation rate of 29% (Figure 3B).

### Pathway analysis of molecular subtypes

**FIGURE 4:**
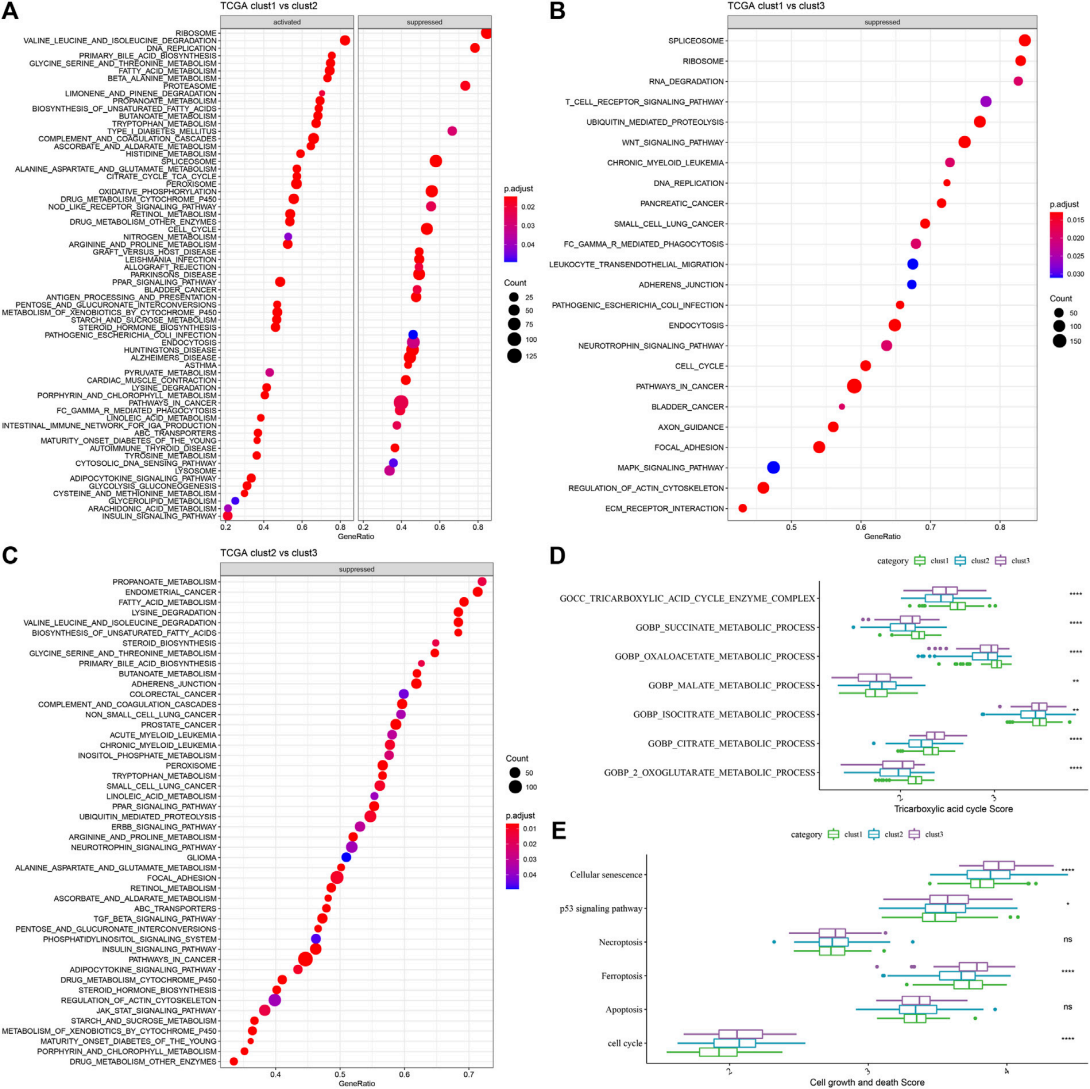
GSEA analysis of the 3 molecular subtypes. **(A)** Clust1 vs. clust2 GSEA analysis results in TCGA-LIHC dataset; **(B)** Clust1 vs. clust3 GSEA analysis results in TCGA-LIHC dataset; **(C)** Clust2 vs. clust3 GSEA analysis results in TCGA-LIHC dataset; **(D)** Comparison of TCA related pathway scores amongst the 3 molecular subtypes identified using the TCGA-LIHC dataset; **(E)** A comparison of cell growth- and cell death-related pathway scores amongst the 3 subtypes in the TCGA-LIHC dataset. (ANOVA, *p < 0.05; **p < 0.01; ***p < 0.001; and ****p < 0.0001).

A comparative analysis of the pathways related to various biological processes in differing molecular subtypes was carried out. The results showed that when clust1 was compared to clust2 of the TCGA-LIHC dataset, the metabolic pathways like the ASCORBATE_AND_ALDARATE_METABOLISM were activated in clust1, while pathways like the SPLICEOSOME were activated in clust2. In clust1 vs. clust3, pathways such as SPLICEOSOME were activated in clust3, while in clust2 vs. clust3, pathways such as FATTY_ACID_METABOLISM were activated in clust3 (Figures 4A–C). Through previous studies, it was found that cuproptosis was related to TCA (Tsvetkov et al., 2022). ssGSEA analysis of the TCA score showed that the three subtypes had significant differences in TCA-linked pathways (Figure 4D). Then, the score of cell growth and death-associated pathways was calculated by the ssGSEA technique. The results showed no significant differences between the 3 subtypes except necroptosis and apoptosis, and there were significant differences among the other four pathways related to cell growth and death (Figure 4E).

### Immune properties displayed by the various molecular subtypes

**FIGURE 5:**
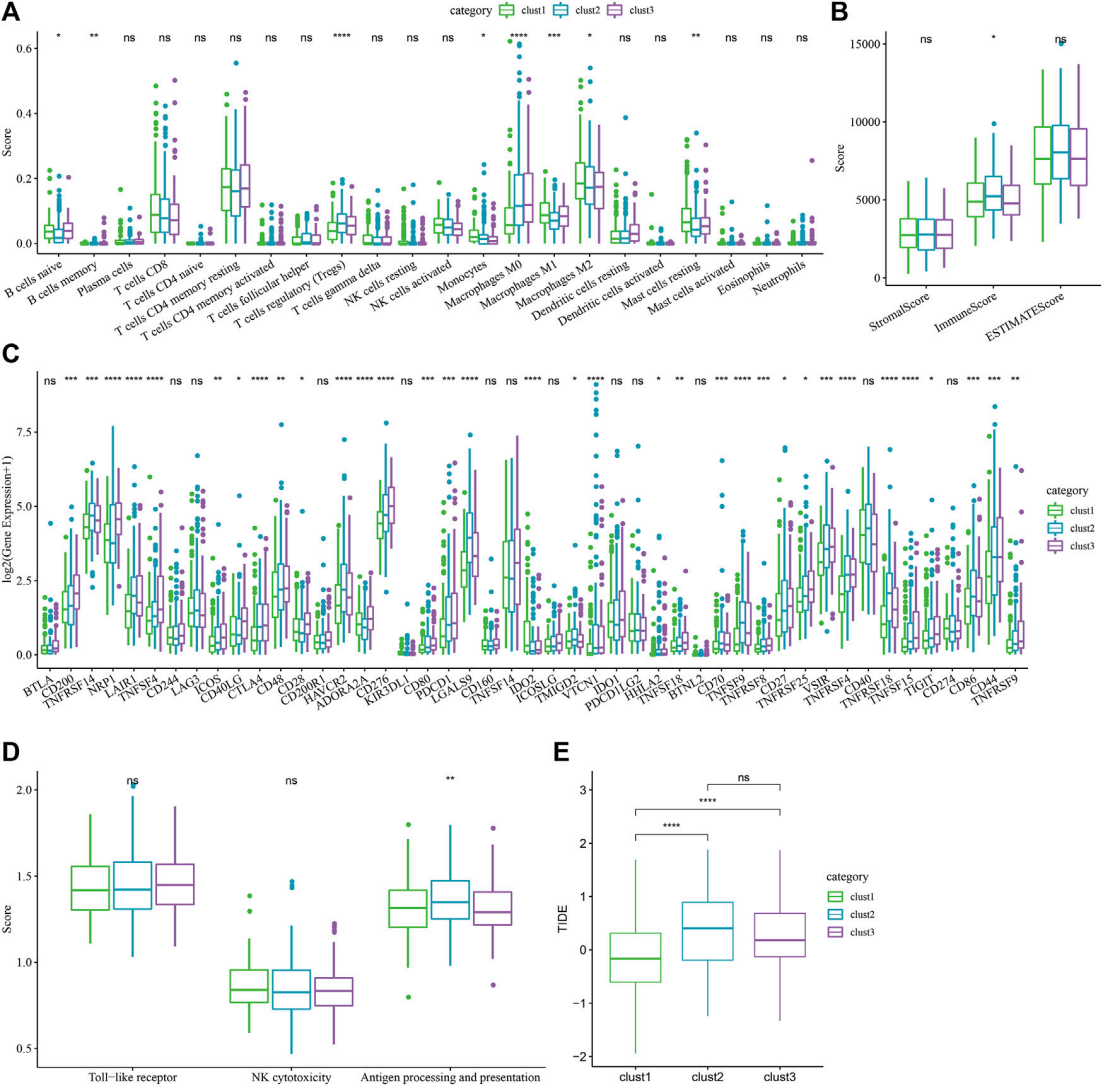
Immune-related characteristics of each cuproptosis subtype. **(A)** The variations in the 22 immune cell scores displayed by the 3 molecular subtypes identified using the TCGA-LIHC dataset; **(B)** The difference of ESTIMATE immune infiltration amongst the 3 molecular subtypes identified using the TCGA-LIHC dataset; **(C)** Immune checkpoints that were expressed differentially by the different groups in the TCGA-LIHC dataset; **(D)** Difference analysis of macrophage participation in related pathways between different groups in the TCGA-LIHC dataset; (ANOVA, *p < 0.05; **p < 0.01; ***p < 0.001; and ****p < 0.0001) **(E)** The difference of TIDE analysis results between different groups in the TCGA-LIHC dataset (Wilcox Test, *p < 0.05; **p < 0.01; ***p < 0.001; and ****p < 0.0001).

For determining the differences present in the immune microenvironment of the patients belonging to various molecular subtypes, the expression level of genes in the immune cells was utilized for assessing the level of infiltration of the immune cells in the TCGA-LIHC dataset. First, the relative quantity of 22 immune cells was determined using the CIBERSORT algorithm. Figure 5A revealed substantial disparities across the different subtypes, associated with 8 immune cell types, including the memory B cells, naive B cells, regulatory T cells, and macrophages (M0, M1, and M2), etc. The immune cell infiltration was also assessed simultaneously using ESTIMATE. The outcomes demonstrated that the three “ImmuneScore” subtypes differed significantly from one another. The “ImmuneScore” of the clust3 subtype having a poor prognosis was lower than that of other subtypes, with low immune cell infiltration (Figure 5B). Further, the sensitivity differences of different molecular subtypes in the TCGA-LIHC dataset to immunotherapy were analyzed. Firstly, the variation in the expression of various immune checkpoints in the different subtypes was compared. The findings showed that these molecular subtypes differentially expressed 34 immune checkpoint genes (Figure 5C). Figure 5A showed that the molecular subtypes particularly showed a differential expression of the macrophages, which are cells that play a vital role in immune regulation, such as Toll-like receptor signaling pathway, and macrophage antigen processing and presentation. And there are FC receptors on the surface of macrophages, which can kill tumor cells through specific antibodies, like the Antibody-Dependent Cell-mediated Cytotoxicity (ADCC) effect (NK-cell mediated cytotoxicity). Therefore, the ssGSEA was used to calculate the immune scores like NK Cytotoxicity Scores, Toll-like Receptor Score, and Antigen Processing and Presentation Score, for every sample. Simultaneously, the ANOVA test found that there were significant differences in macrophages in antigen processing and presentation (Figure 5D). Finally, the TIDE software was employed for analyzing the differences between the different subtypes with regard to immunotherapy. Figure 5E showed that the TIDE score of clust2 and clust3 subtypes in the TCGA queue was higher than the clust1, suggesting that the clust1 subtype had a lower probability of immune escape and showed a higher probability of benefitting from immunotherapy.

### Establishment and validation of clinical prognosis model

Then, the cuproptosis-linked genes, which differed between various subtypes, were identified. Finally, a total of 499 differential genes were chosen for additional analysis, and the results of the differential analysis were shown in the volcanic map (Supplementary Figures S3A–C). Univariate Cox analysis was carried out on 499 differential genes, and a total of 15 genes showing a significant impact on prognosis were identified (*p* < 0.001), including 11 “Risk” and 4 “Protective” genes (Supplementary Figure S3D). Supplementary Figure S3E shows the forest map of univariate Cox analysis of 15 prognosis-related genes.

**FIGURE 6:**
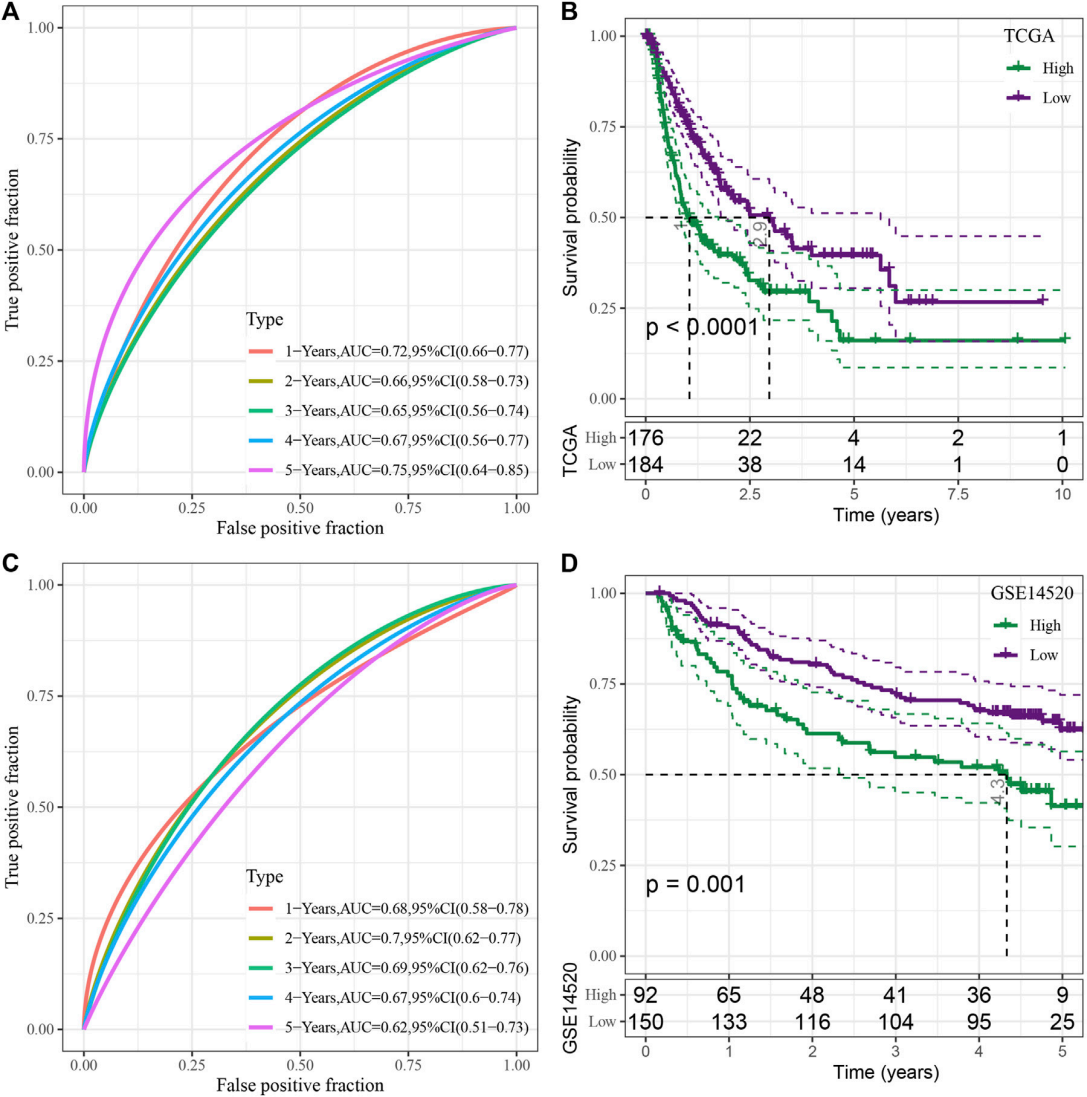
Construction and assessment of the RS model based on the seven cuproptosis-linked genes. **(A)** ROC curve of risk model constructed by seven genes in the TCGA-LIHC dataset; **(B)** KM curve of risk model constructed by seven genes in the TCGA-LIHC dataset; **(C)** ROC curve of risk model constructed by seven genes in the GSE14520 dataset; **(D)** KM curve of the risk model constructed by seven genes in the GSE14520 dataset.

Then, 15 genes were further compressed using the stepwise regression technique, and 7 genes were derived, i.e., KIF2C, PTTG1, CENPM, CDC20, CYP2C9, SFN, CFHR3. The RSs of each sample were calculated through 7 gene expression levels with TCGA-LIHC data as the training data set. Then receiver operating characteristic (ROC) analysis was used for determining the classification efficiency of the prognosis prediction for 1–5 years. Area under the curve (AUC) for 1-, 2-, 3-, 4- and 5-year OS were seen to be 0.72, 0.66, 0.65, 0.67, and 0.75, respectively, wherein the AUC values for 1- and 5-years were >0.7 (Figure 6A). Simultaneously, Z-score conversion was performed on RS. Samples with RS > 0 were classified into the “high-risk” group, while samples with RS < 0 were categorized into the “Low-risk” group, and KM curves were drawn. Results revealed significant differences between both the groups (*p* < 0.0001), and “high” group showed a worse prognosis compared to the “low” group (Figure 6B).

To verify the robustness of the model, the GSE14520 dataset was used to verify by the same method, and ROC curves were used for analyzing the classification efficiency of predicting the prognosis of 1–5 years of OS. The results revealed that the risk model could be effectively developed using the 7 genes. The AUC values for 1–5-year OS were seen to be 0.68, 0.7, 0.69, 0.67, and 0.62, respectively, wherein the AUC values for 2-years were >0.7 (Figure 6C). The same method was used to draw the KM curve, and both the groups showed significant differences (*p* < 0.05). Furthermore, the prognosis of the “low” group was significantly better compared to the “high” group (Figure 6D).

### Performance of the RiskScore in different clinicopathological features and different molecular subtypes

**FIGURE 7:**
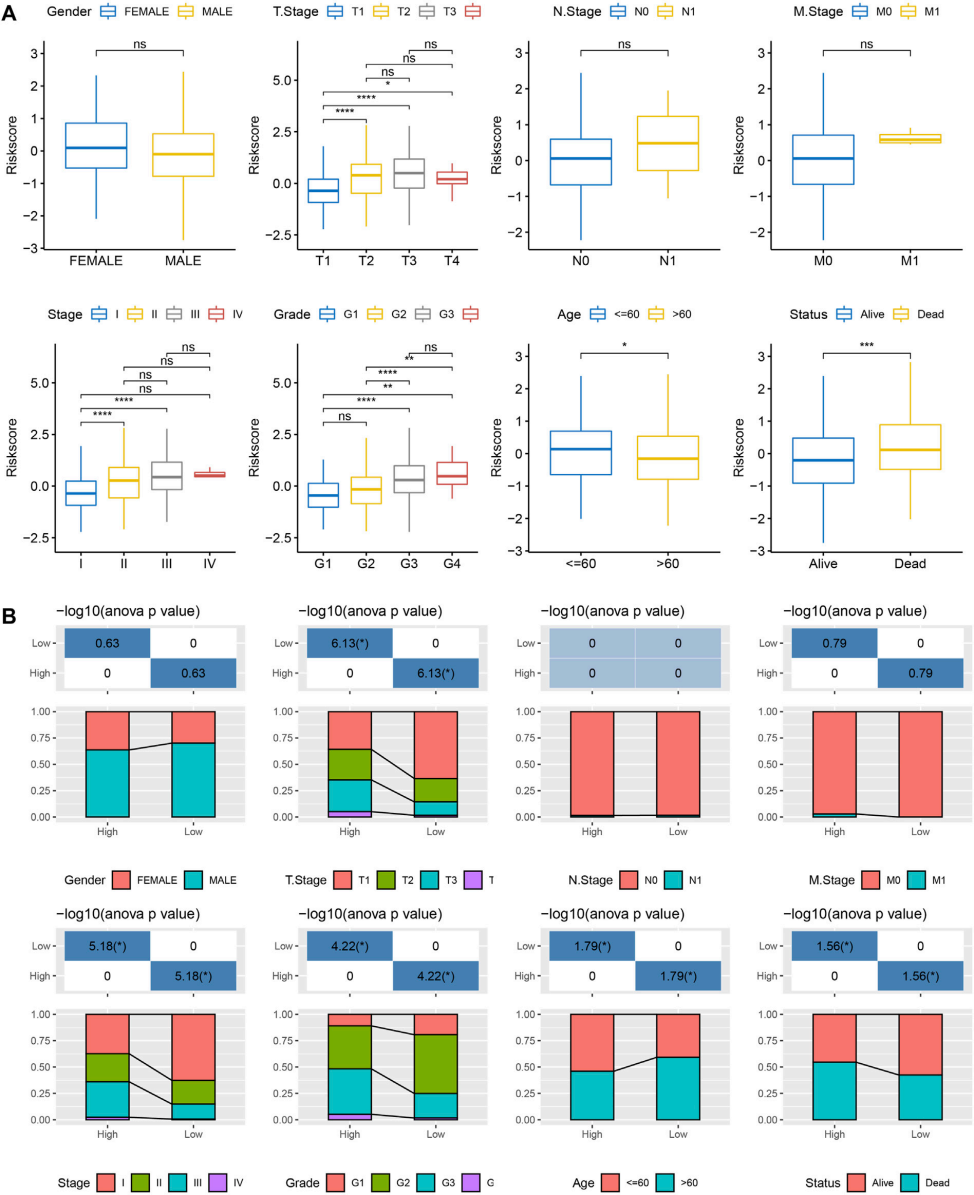
Correlation between RS and clinicopathological features. **(A)** Differences in RSs between different clinicopathological groups in the TCGA-LIHC dataset (Wilcox Test, *p < 0.05; **p < 0.01; ***p < 0.001; and ****p < 0.0001); **(B)** Clinicopathological features between RS groups in the TCGA-LIHC dataset.

For testing the correlation between the RS scores and the clinical characteristics of liver cancer, the difference in RS scoring between different TNM grades and Stage clinical grades in the TCGA-LIHC dataset was evaluated. The results implied that samples with higher clinical grades had higher RS (Figure 7A). Simultaneously, the clinicopathological differences between the RS groups in the TCGA-LIHC dataset were compared and significant differences were noted in the distribution of T-stage, Stage, Grade, Age, and Status between both the groups. “High” showed a higher clinical grade, and a greater number of patients died in the “high” group, which was in agreement with poor prognosis (Figure 7B).

### Differences in immune characteristics and immunotherapy among RiskScore groups

**FIGURE 8:**
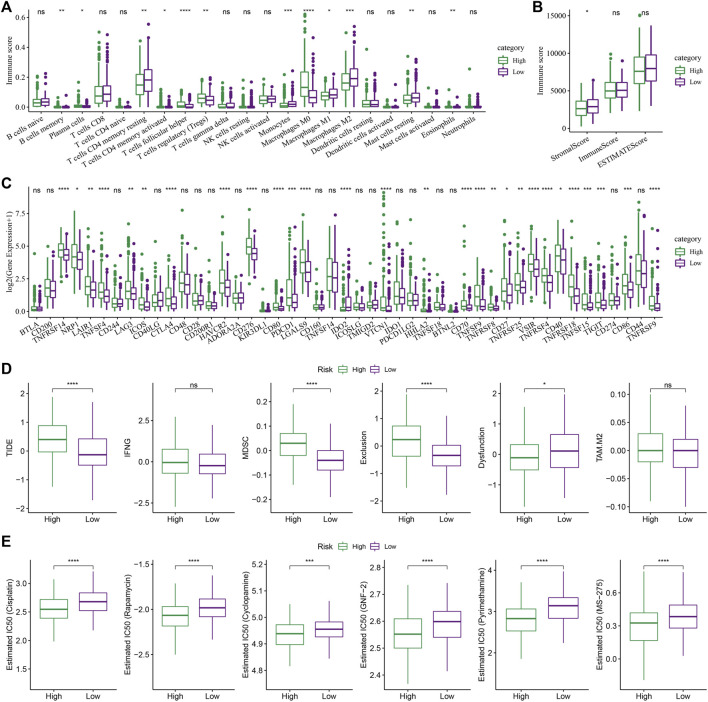
The role of the RS model in predicting the benefits of immunization/chemotherapy. **(A)** Variations in the 22 immune cell scores amongst the different risk groups identified using the TCGA-LIHC dataset; **(B)** Differences in immune and matrix scores amongst the different risk groups in the TCGA-LIHC dataset; **(C)** Immune checkpoints differentially expressed between various groups in the TCGA-LIHC dataset; **(D)** The results of TIDE analysis among different groups in TCGA-LIHC dataset were different; **(E)** Box plots of the calculated IC50 for the drug in TCGA-LIHC dataset (Wilcox test, *p < 0.05; **p < 0.01; ***p < 0.001; and ****p < 0.0001).

The changes in the relative abundance of 22 different immune cell types in the high-RS and low-RS groups were examined in order to better understand the differences in the immunological microenvironment of patients in the RS group. The results showed that there were notable differences between the RS-high and -low groups in 12 different immune cell types, including plasma cells, macrophages (M0, M1, M2), memory B cells, etc. (Figure 8A, Wilcox.test). Additionally, the immune cell invasion was also evaluated using ESTIMATE (Wilcox.test). The findings demonstrated a statistically significant difference in the “ImmuneScore” values between the 2 groups. With a higher immune cell infiltration, the “ImmuneScore” in the “low” group was seen to be higher compared to that in the “high” group (Figure 8B).

Then the sensitivity difference of immunotherapy between the low and high-risk groups in the TCGA-LIHC dataset was analyzed. Firstly, the differences in the expression of the immune checkpoints between various checkpoints were compared. The results indicated that 28 immune checkpoint genes were expressed differentially between both the groups (Figure 8C, Wilcox.test).

Furthermore, the potential clinical effects of immunotherapy in the high- and low-RS groups in the TCGA-LIHC dataset, were analyzed, using the TIDE software. The analysis revealed significant differences in the MDSC, TIDE, Exclusion, and Dysfunction scores. It was concluded that the High-RS group showed higher scores than those shown by the Low-RS group (Figure 8, Wilcox.Test). In addition, the response degree of the high-risk and low-risk groups to traditional chemotherapy drugs was analyzed. It was found that there were significant differences among five traditional drugs, cisplatin, rapamycin, cyclopamine, GNF-2, and pyrimethamine, and the “high” group was more sensitive to these traditional drugs (Figure 8E, Wilcox.test).

### Abnormal performance of RiskScore in tricarboxylic acid pathway

**FIGURE 9:**
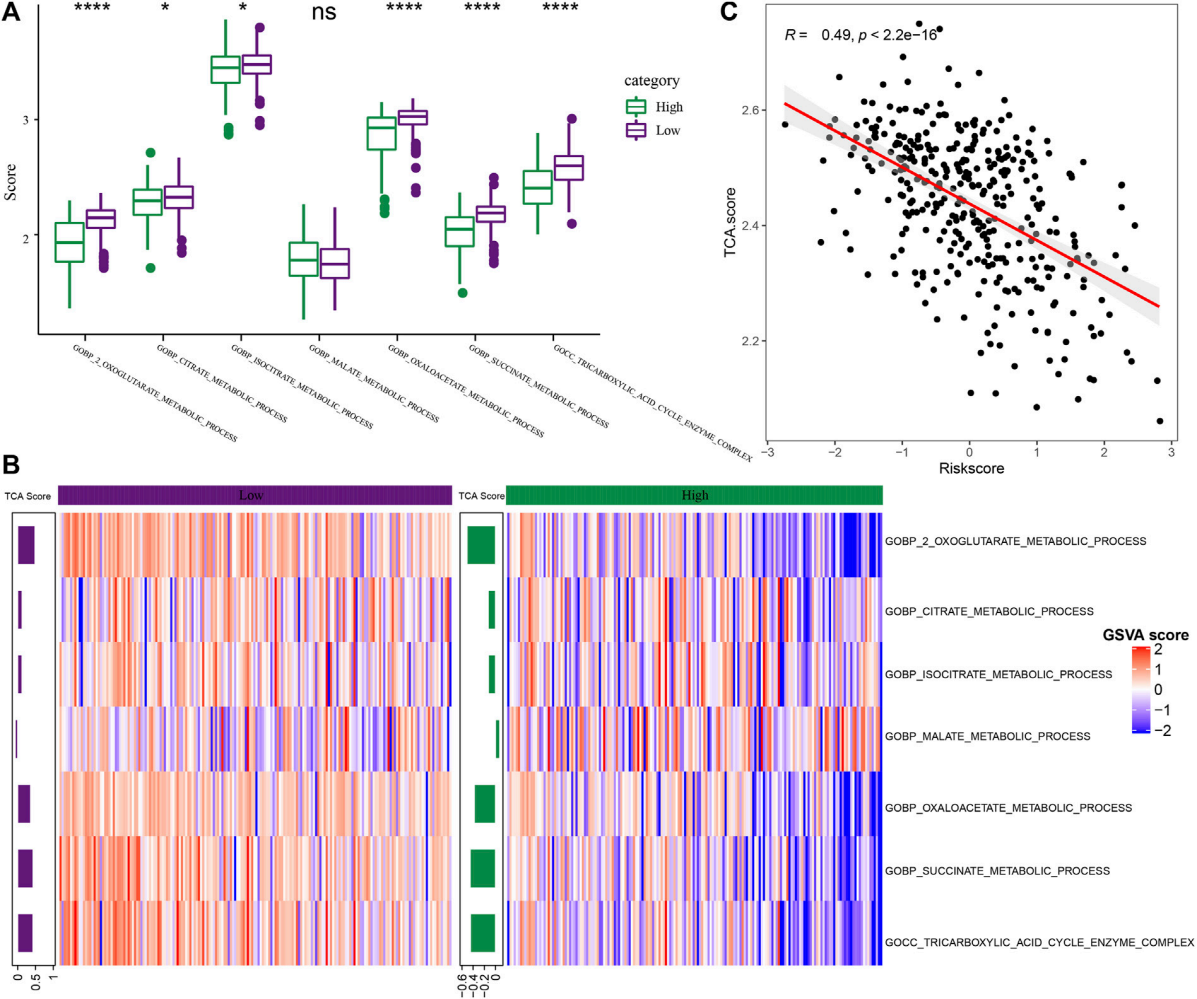
Differences in pathway characteristics among different RS groups. **(A)** The box plot of TCA-related pathway scores in high-risk and low-risk groups in the TCGA dataset (Wilcox.test, *p < 0.05; **p < 0.01; ***p < 0.001; and ****p < 0.0001); **(B)** The heat map of TCA related pathway scores in the high-risk and low-risk groups in the TCGA-LIHC dataset; **(C)** The scatter diagram of correlation analysis between RS and TCA scores in TCGA-LIHC dataset.

Further, the performance of RS in TCA-related pathways was compared. As shown in Figure 9A, it was noted that the score of the TCA-linked pathway in the “low” group was higher. Both the groups showed significant differences with regards to different pathways like the COBP_CITRATE_METABLIC_PROCESS, COBP_2_OXOGLUTARATE_METABLIC_PROCESS, COBP_OXOGLUTARATE_

METABLIC_PROCESS, COBP_SUCCINATE_METABLIC_PROCESS, and COBP_TRICARBOXYLIC_ACID_CYCLE_ENZYME_COMPLEX pathways. Then, the score of TCA-related pathways for every patient included in the TCGA-LIHC dataset was z-scored based on the samples in the heat map for determining the difference in the scores of the related pathways in the expression pathway of the high-risk and low-risk groups, and the TCA score of “low” group was higher (Figure 9B). The average value was calculated as the TCA score through the score of TCA-related pathways. Through analysis, it was found that the TCA score was significantly and negatively related to RS (R = 0.49, *p* < 2.2e-16) (Figure 9C, Spearman).

### RiskScore combined with clinicopathological features to further improve the prognostic model and survival prediction

**FIGURE 10:**
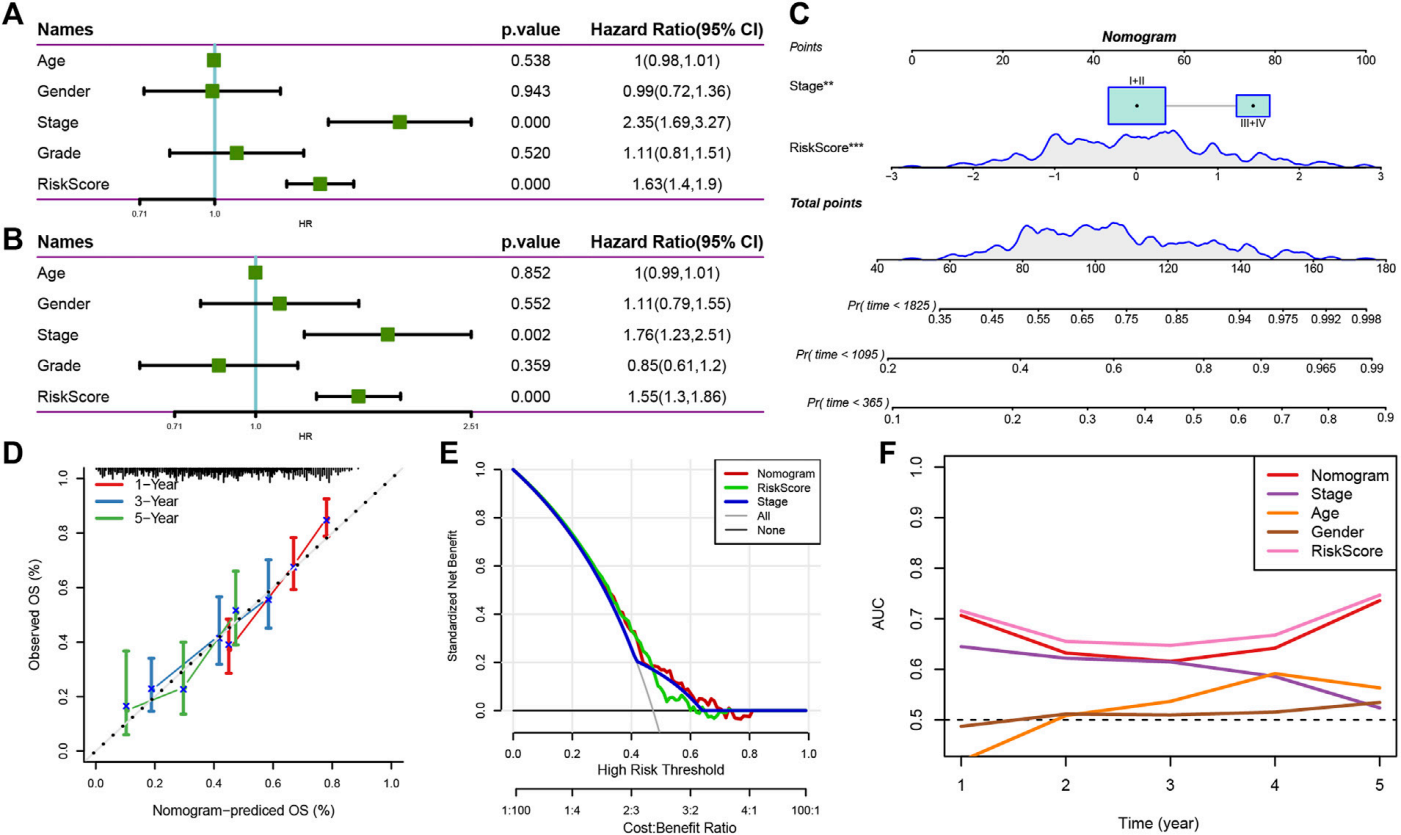
Nomogram of RS combined with clinical pathological characteristics. **(A,B)** Univariate and Multivariate Cox regression analysis of the RS and clinicopathological features; **(C)** Nomograph model; **(D)** Calibration curve of nomograph in 1, 3, and 5 years; **(E)** Decision curve of nomograph; **(F)** When compared to a few other clinicopathological characteristics, the nomogram displayed a good capacity for OS prediction.

Clinicopathological characteristics and RS were analyzed using the Univariate and Multivariate Cox regression analysis, and the results revealed that Stage and RS were the most important predictive markers (Figures 10A,B). RS and Stage were coupled to create a nomogram in order to evaluate the risk assessment and the survival probability of the liver cancer patients (Figure 10C). According to the model’s findings, RS had the biggest influence on the survival rate prediction. Then, the calibration curve was utilized to assess the model’s predictability, as illustrated in Figure 10D. It can be seen that the three calibration points for the prediction calibration curves for 1, 3, and 5 years were close to the reference curve, indicating that the nomogram performed well in terms of prediction. Additionally, decision curve analysis (DCA) was utilized to investigate the model’s dependability. It is evident that the advantages of RS and nomogram were much greater than those of the extreme curve. The nomogram and RS demonstrated the highest capacity to predict survival when compared to other clinicopathological characteristics (Figures 10E,F).

## Discussion

The long-term efficacy of HCC treatment is still a significant problem in clinical practice because of the complexity of the TME and heterogeneity of HCC. The selection of the best course of treatment and action must be categorized and improved. In HCC, many transcriptome-based classifications are extensively used. Li et al. identified 2 novel molecular subgroups in liver cancer based on genes associated with ferroptosis and created a ferroptosis-associated prognostic RS model made up of six genes that can be used to predict outcomes and identify the cancer patients who would respond well to immunotherapy. Three molecular subtypes of HCC were discovered by Wang et al., each with a unique prognosis and metabolic profile. Lin et al. found 18 lncRNAs and 2 molecular subtypes in HCC with specific immune dysfunction that present distinct prognostic characteristics and immunological characteristics, which aids in understanding the function of lncRNA and motivates the discovery of immunotherapy targets. In this study, the HCC molecular subtypes were detected, from the cuproptosis perspective, since one cannot ignore the regulatory impact of cuproptosis.

From the literature review, 13 genes associated with cuproptosis were obtained. After analyzing the differences in the mRNA expression of cuproptosis-related genes between the primary tumor samples and adjoining normal tissue samples, it was noted that a majority of these variations were statistically significant. HCC patients were categorized into three categories depending on the consistent clustering of 13 cuproptosis-related gene expression profiles. The three subtypes had distinct prognosis characteristics, according to the prognostic analysis. The prognosis for Clust3 was the worst, followed by Clust2 and Clust1, while Clust1 showed the best prognosis. Additional examination of the clinicopathological traits of the various subtypes revealed that the Clust3 subtype showed a late clinical T stage and Stage, in addition to a higher Grade. All these findings were in agreement with its poor prognosis.

Further, the genomic variations displayed by the 3 molecular subtypes, identified using the TCGA-LIHC dataset were analyzed. The results implied that the clust1 subtype showed a lower Aneuploidy Score and Homologous Recombination Defect. Additionally, after comparing the correlation between the 5 existing immune molecular subtypes and the 3 molecular subtypes defined in this study, it was noted that among clust3 and clust2 subtypes with a poor prognosis had a low proportion of C4 subtype (lymphocyte depleted subtype) and a high percentage of C1 subtype (wound healing subtype), which was consistent with the prognosis of immune molecular subtypes. Then, the variations in the gene mutations existing between the different molecular subtypes were also identified, and significant differences were noted in the mutation frequencies of TP53, TTN, MUC16, and other genes among the 3 molecular subtypes, and clust1 had a higher mutation frequency of TP53.

Recent reports have stated that the concentration of the copper ions in the tumor tissues and serum of cancer patients was significantly higher than those of healthy patients (Blockhuys et al., 2017). Previous studies have shown that cuproptosis takes place by the direct combination of copper ions and the fatty acylated components present in the TCA cycle. This leads to the accumulation of the fatty acylated proteins and subsequent loss of the iron-sulfur cluster proteins, which leads to protein toxic stress and eventually cell death. Therefore, cuproptosis is closely related to the TCA cycle (Tsvetkov et al., 2022). TCA is seen to play a significant role in cellular energy metabolism and it is also responsible for the onset of numerous diseases, like tumors. At present, mutations and abnormal expression of TCA key genes have been found in tumors, which are significantly related to tumorigenesis and progression. The liver is an important digestive organ in the human body. The metabolic process of three major nutrients occurs actively in the liver. HCC is usually accompanied by the TCA cycle reprogramming, which regulates energy production through the TCA cycle, which ensures the survival of the tumor cells even in difficult conditions like hypoxia, nutrient deficiency, and finally, escaping the immune system (Ferrarini et al., 2019; Du et al., 2022). The score of TCA related pathway was calculated by the ssGSEA method. Significant differences were noted in the 3 molecular subtypes, with regard to the TCA cycle. Cell death is an essential and fine-tuning process, which is crucial to eliminating damaged and redundant cells. Many forms of programmed and non-programmed cell death have been identified, including apoptosis, ferroptosis, and necroptosis (Moujalled et al., 2021). This study could not detect any significant differences between the 3 molecular subtypes, except in necroptosis and apoptosis, and there were significant differences in the other four pathways related to cell growth and death.

The metabolic environment can change the immune response in the liver and make tumor cells immune escape. In addition, metabolic rearrangement of immune cells can cause abnormal self-function (Li et al., 2021). TME is a crucial intrinsic factor in the emergence, growth, invasion, and metastasis of liver cancer. The findings of this study showed that different molecular subtypes exhibited varying degrees of immune cell infiltration in the immunological microenvironment of different patients. Additionally, the “ImmuneScore” of the clust3 subtype, which has a poor prognosis due to the relatively low immune cell infiltration, was lower compared to that of other subtypes. Additionally, the immunotherapy sensitivity variations of several molecular subtypes in the TCGA-LIHC dataset were examined. The majority of immunological checkpoint genes were discovered to express differently in various subtypes. In each of the three molecular categories, the macrophages showed a significant difference. Macrophages are crucial for immunological regulatory processes such as the processing and presentation of antigens and the Toll-like receptor signaling pathway. Additionally, macrophages have FC receptors on their surface, which when activated by a specific antibody can cause an ADCC effect (NK cell-mediated cytotoxicity) that kills tumor cells (Xing et al., 2020). Through our analysis, macrophages had significant differences in the antigen processing and presentation pathway. Lu et al. (2022) showed that PD-L1 positive host macrophages, representing the main cell source of PD-L1 in HCC, showed HLA-DR^high^CD86^high^ glycolysis phenotype, significantly produced anti-tumor IL-12p70, and polarized through internal glycolysis metabolism. These results implied that the macrophages play a vital role in the onset of liver tumors, and the distribution differences between the three subtypes can provide a basis for tumor treatment. Further, by analyzing the sensitivity differences among different subtypes in immunotherapy, it was found that the clust2 and clust3 subtypes in the TCGA-LIHC dataset showed higher TIDE scores than those presented by clust1, suggesting that the clust1 subtype had a lower probability of immune escape and greater likelihood of benefiting from immunotherapy.

Then, a total of 15 genes among the three subtypes that had a significant impact on prognosis were identified, and the prognostic risk model was constructed by KIF2C, PTTG1, CENPM, CDC20, CYP2C9, SFN, CFHR3, and seven genes was obtained by Lasso regression and AIC algorithm. Kinesin Family member 2C (KIF2C) belongs to the kinesin 13 family, and is an M-kinesin, which is overexpressed in many human tumors. In their study, Wei et al. (2021) observed that KIF2C was overexpressed in HCC and was related to several aggressive malignancies that activate the Wnt/β-catenin signaling pathway and was also involved in the HCC progression as it interacted with TBC1D7 in mTORC1 signaling. Cho-Rok et al. (2006) found that Pituitary Tumor Transforming Gene 1 (PTTG1) was overexpressed in many types of human cancers. Furthermore, results indicated that when the PTTG1 gene was silenced, it inhibited the growth of the liver cells *in vivo* and *in vitro*. Studies have shown that centromeric protein M (CENPM) is closely related to the development of HCC. The up-regulation of CENPM promotes hepatocarcinogenesis through a variety of mechanisms and could be considered a new probable biomarker and a clinical therapeutic target for HCC (Xiao et al., 2019). Studies have found that CDC20 regulates the process of the cell cycle mainly by targeting the destruction of key substrates. In HCC, CDC20 binds to the Destruction box (D-box) motif in oxygen-dependent Prolyl Hydroxylase 3 (PHD3) to promote its polyubiquitination and degradation and is seen to play a vital role in HCC development by controlling PHD3 (Shi et al., 2021). Nizamuddin et al. found that cytochrome-P450-2C9 (CYP2C9) has genetic diversity. This gene metabolizes many drugs and is overexpressed in the human liver (Nizamuddin et al., 2021). Sulforaphane (SFN) plays an epigenetic regulatory role by inhibiting histone deacetylase (HDAC) and affects the activity of carcinogenic transcription factors through the methylation of its binding site motif, which provides insights into the chemopreventive molecular effects of SFN in HepG2 cells. It is a valuable natural cancer treatment method (Dos Santos et al., 2020). Complement factor H-related 3 (CFHR3) is a protein-coding gene that plays a role in various diseases. Liu et al. (2020) found through bioinformatics analysis that CFHR3 is a novel prognostic biomarker and therapeutic target for determining HCC.

Further, the relationship between RS scoring and clinical characteristics of liver cancer was analyzed. It was found that the samples with higher clinical grades had higher RSs. A comparison of the different immune microenvironments in the patients belonging to differing RS groups showed that the “low” group presented a high infiltration of immune cells, and a majority of the Immune checkpoint genes were expressed differentially in both groups. In addition, by analyzing the sensitivity difference between RS group to treatment, it is noted that the “high” group showed a higher TIDE score compared to the “low” group, indicating that the likelihood of immune escape in the high-risk group was higher compared to the low-risk group, and the high-risk group patients were less likely to be benefitted from immunotherapy. However, the “high” group was more sensitive to these traditional drugs. This result can provide a reference for personalized treatment of patients. Simultaneously, the performance of RS in TCA-related pathways was compared. The results in this study showed that the low-risk group showed higher TCA-related pathways scores, and the TCA scores were seen to be significantly negatively related to the RS, which was consistent with the results of subtype typing, and TCA scores with poor prognosis were higher. Finally, the clinical characteristics that showed significant differences during the Univariate and Multivariate Cox regression analysis, Stage, and RS were used for constructing a novel nomogram. Analyzing the calibration and the decision curves indicated that the model showed a higher prediction accuracy and survival prediction capacity. Additionally, the cuproptosis-linked genes were chosen as the target gene, which was essential for the onset, development, diagnosis, and treatment of HCC. The nomogram model constructed in this study could be used as the basis for deriving an individualized treatment plan for HCC patients.

This study provides novel insights into the personalized clinical treatment planning for HCC patients, however, it does have a few limitations. First of all, our research only includes bioinformatics analysis and lacks the verification of experimental clinical samples. In addition, the study was carried out using a retrospective design instead of using a prospective design. However, this analysis was carried out using 2 independent datasets, so the results are still acceptable and reliable. It can be concluded that prospective clinical trials and an investigation into the mechanisms involved need to be carried out for verifying the results noted in the study.

## Conclusion

To conclude, this study presented 3 molecular subtypes that were associated with cuproptosis in liver cancer. These 3 molecular subtypes showed a heterogeneity in their pathological features, prognosis, pathway, and immune characteristics. Thereafter, a classifier known as the prognostic risk model associated with cuproptosis was constructed and verified. The model has strong stability, is independent of the clinical and pathological characteristics, and plays a stable prediction efficiency in independent data sets. The model has high prediction accuracy and survival prediction ability, which could be used for predicting prognosis and selecting the immunotherapy that was best suited for the patients. These results could help in developing a precise and individualized treatment strategy for clinical HCC patients.

## Data Availability

Publicly available datasets were analyzed in this study. The names of the repository/repositories and accession number(s) can be found in the article/Supplementary Material.
