# Supplementary figures and images for: Risk model of hepatocellular carcinoma based on cuproptosis-related genes
Source: Front Genet. 2022 Sep 15;13:1000652. doi: 10.3389/fgene.2022.1000652 (PMC9521278; doi:10.3389/fgene.2022.1000652)

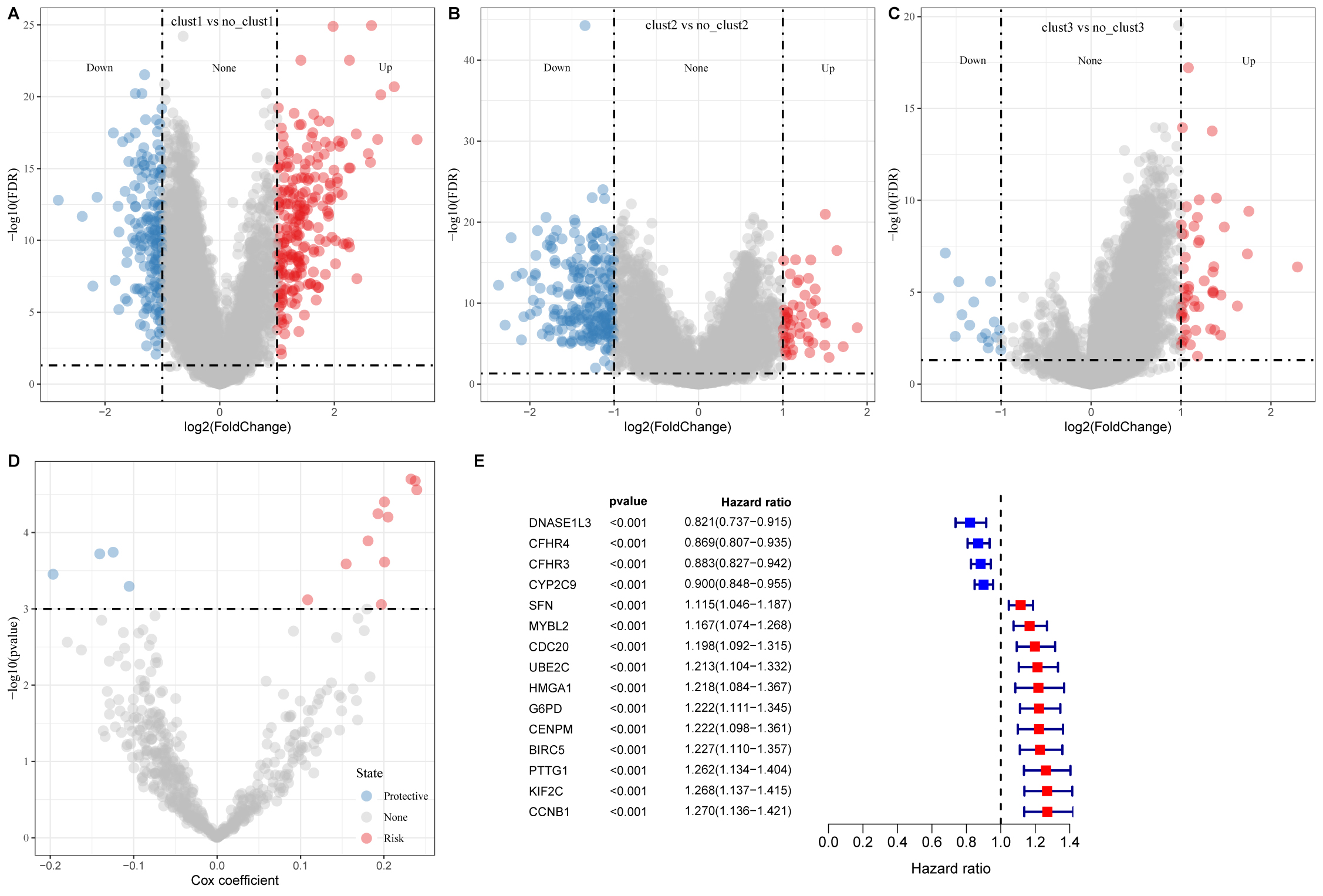

Supplement: Supplementary file 1 [file Image3.JPEG]

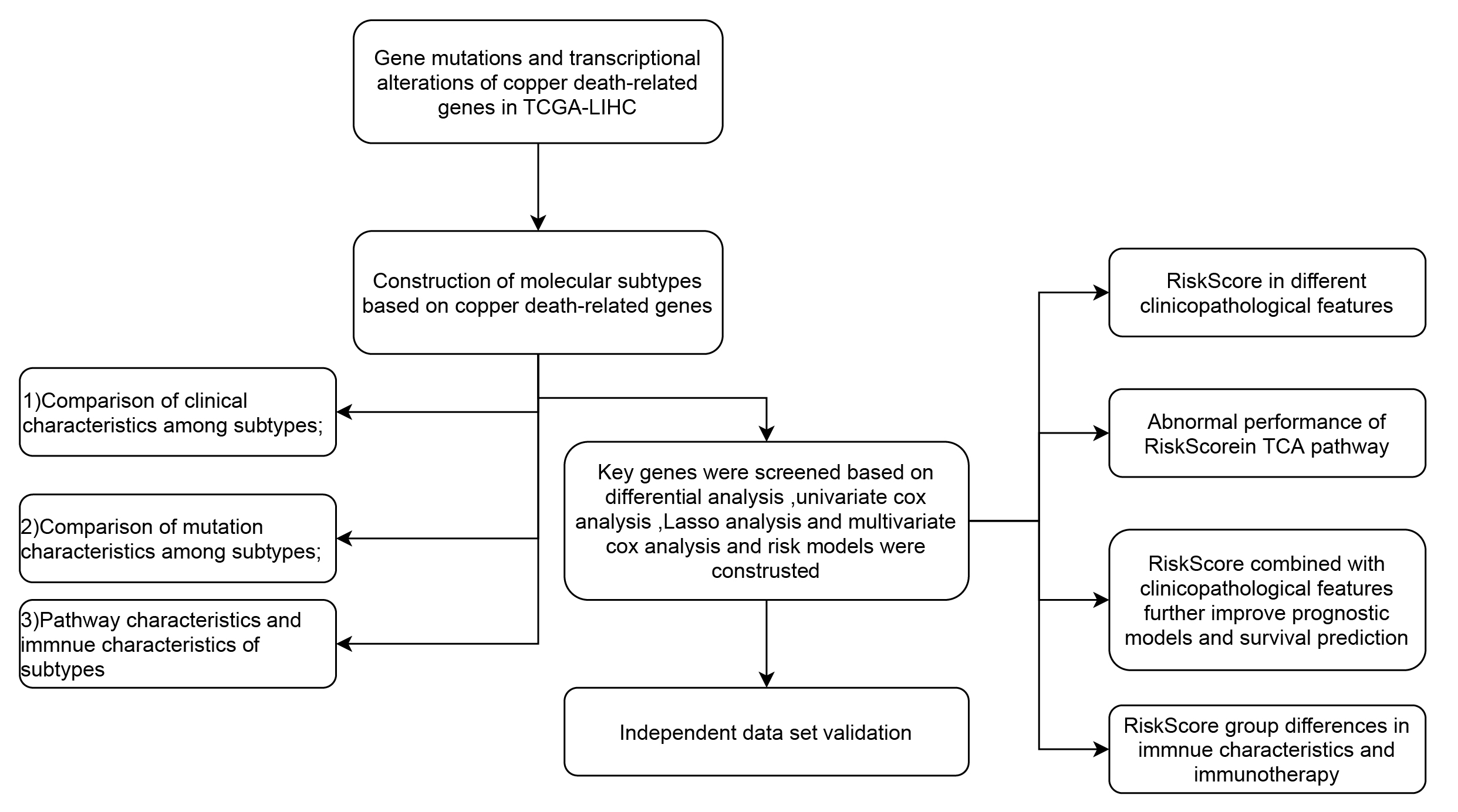

Supplement: Supplementary file 3 [file Image1.JPEG]

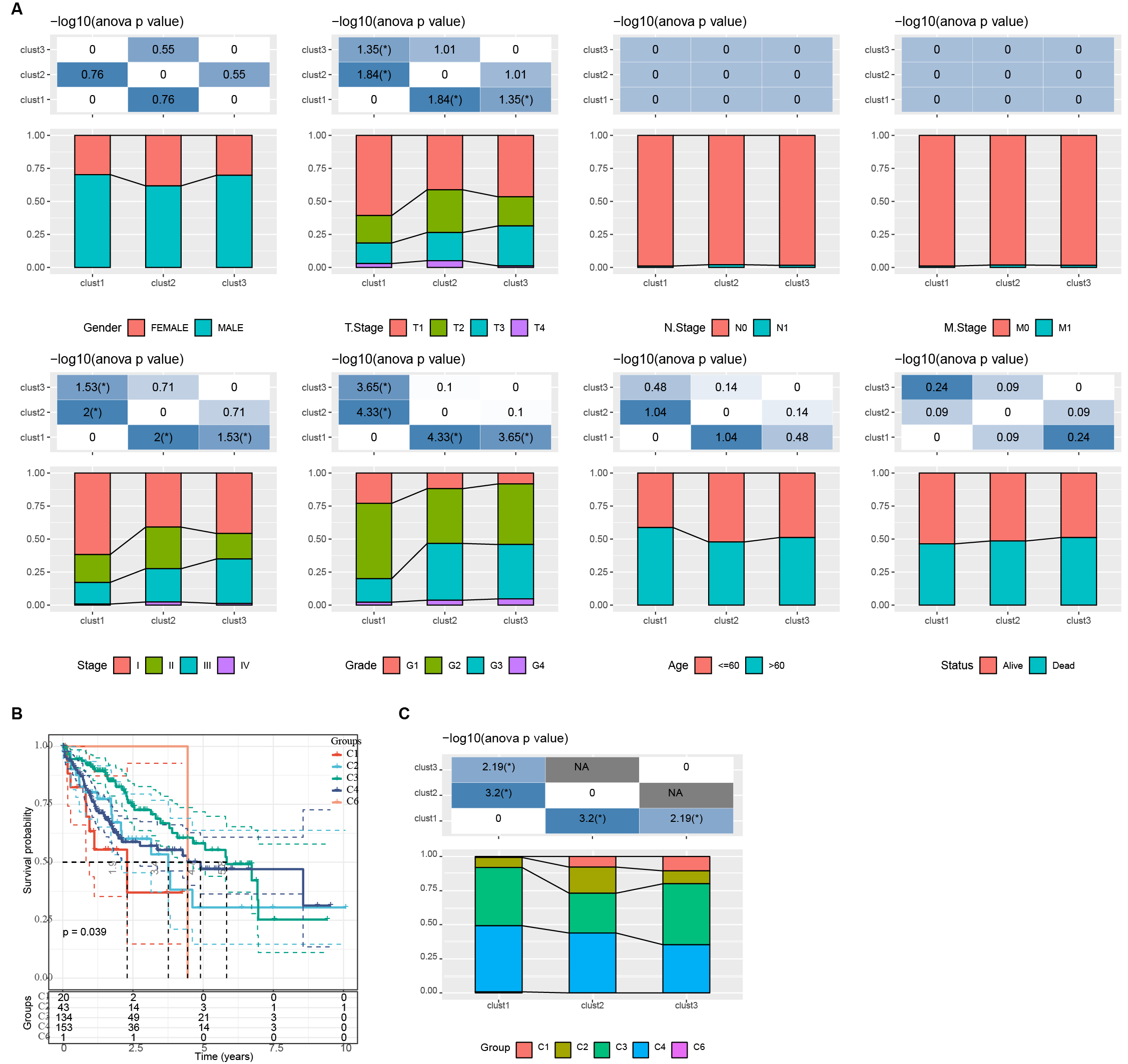

Supplement: Supplementary file 4 [file Image2.JPEG]
