# Supplementary material for: Risk model of hepatocellular carcinoma based on cuproptosis-related genes
Source: Front Genet. 2022 Sep 15;13:1000652. doi: 10.3389/fgene.2022.1000652 (PMC9521278; doi:10.3389/fgene.2022.1000652)
Supplement: Supplementary file 2 [file Table1.DOCX]

| Copper death-related genes | | | |
| --- | --- | --- | --- |
| FDX1 | LIPT1 | LIAS | DLD |
| DBT | GCSH | DLST | DLAT |
| PDHA1 | PDHB | SLC31A1 | ATP7A |
| ATP7B |  |  |  |
