# Supplementary material for: Risk model of hepatocellular carcinoma based on cuproptosis-related genes
Source: Front Genet. 2022 Sep 15;13:1000652. doi: 10.3389/fgene.2022.1000652 (PMC9521278; doi:10.3389/fgene.2022.1000652)
Supplement: Supplementary file 5 [file Table2.DOCX]

Supplementary materials

Figure S1: The work flow of this study.

Figure S2: (A) Clinical information distribution of molecular subtypes in TCGA-LIHC dataset; (B) Distribution of the five immune subtypes previously studied in the three molecular subtypes in this study; (C) Survival curve of five immune molecular subtypes. * P<0.05.

Figure S3: Identification of key genes of cuproptosis phenotype. (A) Difference analysis volcanic map of clust1 vs no_clust1 in TCGA-LIHC queue; (B) Difference analysis volcanic map of clust2 vs no_clust2 in TCGA-LIHC queue; (C) Difference analysis volcanic map of clust3 vs no_clust3 in TCGA-LIHC queue; (D) 499 promising candidates were detected amongst the DEGs; (E) The forest map of 15 prognosis-related genes.
