# Supplementary material for: Risk model of hepatocellular carcinoma based on cuproptosis-related genes
Source: Front Genet. 2022 Sep 15;13:1000652. doi: 10.3389/fgene.2022.1000652 (PMC9521278; doi:10.3389/fgene.2022.1000652)
Supplement: Supplementary file 6 [file Table3.DOCX]

For the data analyzed in this study please see:

https://www.jianguoyun.com/p/DaLwgxAQharcChjQ48wEIAA
